# Slow train coming: an anti-CCN2 strategy reverses a model of chronic overuse muscle fibrosis

**DOI:** 10.1007/s12079-020-00568-1

**Published:** 2020-05-14

**Authors:** Andrew Leask

**Affiliations:** School of Dentistry, University of Saskatchewan, 105 Wiggins Road, Saskatoon, SK Canada

## Abstract

One of the first targets proposed as an anti-fibrotic therapy was CCN2. Proof of its involvement in fibrosis was initially difficult, due to the lack of appropriate reagents and general understanding of the molecular mechanisms responsible for persistent fibrosis. As these issues have been progressively resolved over the last twenty-five years, it has become clear that CCN2 is a bone fide target for anti-fibrotic intervention. An anti-CCN2 antibody (FG-3019) is in Phase III clinical trials for idiopathic pulmonary fibrosis and pancreatic cancer, and in Phase II for Duschenne’s muscular dystrophy. An exciting paper recently published by Mary Barbe and the Popoff group has shown that FG-3019 reduces established muscle fibrosis (Barbe et al., FASEB J 34:6554–6569, 2020). Intriguingly, FG-3019 blocked the decreased expression of the anti-fibrotic protein CCN3, caused by the injury model. These important data support the notion that targeting CCN2 in the fibrotic microenvironment may reverse established fibrosis, making it the first agent currently in development to do so.

As early as the mid-1990s, CCN2, formerly referred to as connective tissue growth factor (Perbal et al., 2018), was proposed to be an essential mediator of fibrosis and, hence, a target for anti-fibrotic drug intervention (Grotendorst, 1997). Not long after, FibroGen identified an antibody, FG-3019, that recognized CCN2 and was selected for (pre) clinical development, even though the molecular mechanism underlying the pro-fibrotic action of CCN2 and, indeed of this antibody, was unclear (Leask, 2020). This antibody, and the use of conditional CCN2 knockout mice, has conclusively shown that CCN2 is a central mediator of fibrosis, in an albeit cell-type specific fashion, in every fibrotic model thus far examined (Leask, 2020). CCN2 appears to be essential for myofibroblast activation from progenitor cells, in fibrotic, but not wound repair, models by acting downstream of the crucial pro-adhesive signaling pathway (Tsang et al., 2020). FG-3019 is currently being fast-tracked by the US FDA as a therapeutic drug for idiopathic pulmonary fibrosis and Duchenne’s muscular dystrophy (NCT01262001, NCT01890265, NCT02606136) (Richeldi et al., 2020; Leask, 2019, 2020).

In fibrotic conditions, once deposited and cross-linked, the extracellular matrix becomes resistant to degradation (Mahdy, 2019). Moreover, myofibroblasts, the effector cell of fibrosis, appear to persist as they are stably differentiated from progenitor cells (Tsang et al., 2020). Collectively, these features make development of treatments that reverse fibrosis problematic.

Earlier studies by the Popoff laboratory used a rat model of overuse injury to show that FG-3019 blocked the early progression of chronic overuse-induced muscle fibrosis and the associated declines of grip strength (Barbe et al., 2019). Recently, the same group examined if this antibody could alleviate established skeletal muscle fibrosis in the same rat model (Barbe et al., 2020). They reported that FG-3019 decreased collagen and alpha-smooth muscle actin expression and the reduction in grip strength caused by overuse injury. Elevations in ERK phosphorylation were also suppressed. CCN1 was not induced in response to injury, nor was it  suppressed by FG-3019.

Expression of the anti-fibrotic CCN family member CCN3, which has anti-fibrotic effects and is expressed reciprocally to CCN2 (Riser et al., 2015), was reduced in response to overuse injury. This effect was blocked by FG3019, suggesting that FG-3019 may act by blocking the fibrosis-associated decline in CCN3 expression.

In conclusion, targeting CCN2 with FG-3019 may be a viable approach to reverse established fibrosis.
